# Modulation of the Gut Microbiota by Fufang-Zhenzhu-Tiaozhi Capsule Attenuates Hypertension Induced by a High-Fructose and High-Salt Diet

**DOI:** 10.3389/fcimb.2022.854849

**Published:** 2022-06-29

**Authors:** Zhe Chen, Bin Yang, Zhen Wang, Xianglu Rong, Qing Zhu, Jiao Guo

**Affiliations:** ^1^ Science and Technology Innovation Center, Guangzhou University of Chinese Medicine, Guangzhou, China; ^2^ Guangdong Metabolic Disease Research Center of Integrated Chinese and Western Medicine, Guangdong Pharmaceutical University, Guangzhou, China; ^3^ Key Laboratory of Glucolipid Metabolic Disorder, Ministry of Education of China, Guangzhou, China; ^4^ Guangdong TCM Key Laboratory for Metabolic Diseases, Guangdong Pharmaceutical University, Guangzhou, China; ^5^ Institute of Chinese Medicine, Guangdong Pharmaceutical University, Guangzhou, China

**Keywords:** hypertension, gut microbiota, metabolic disorder, Fufang-Zhenzhu-Tiaozhi capsule, fecal microbiota transplantation

## Abstract

Hypertension is frequently comorbid with the disorders of glucose and lipid metabolism. The increased intakes of fructose and salt contribute to the development of hypertension and related metabolic disorders, which are closely associated with gut dysbiosis. Fufang-Zhenzhu-Tiaozhi capsule (FTZ), a traditional Chinese patent medicine commonly used in clinical practice, has recently emerged as a promising drug candidate for metabolic diseases. In this study, FTZ treatment is identified as attenuating blood pressure increase and improving the metabolism of lipid and uric acid in high-fructose and high-salt (HFS) diet-fed rats. FTZ also substantially alleviated renal fibrosis and the mRNA expression of inflammation cytokines, NADPH oxidases, and the renin–angiotensin system in the renal cortex. 16S rRNA sequencing of fecal samples revealed that FTZ restored HFS-induced gut dysbiosis, seen as increased intestinal microbial richness and diversity. Furthermore, fecal microbiota transplantation also achieved similar therapeutic effects and alterations in gut microbiota profile induced by FTZ. Taken together, this study highlights the efficacy of FTZ in attenuating HFS-induced hypertension and related metabolic disorders and renal injury. The antihypertensive effect is associated with the modulation of gut microbiota.

## 1 Introduction

Hypertension, a common chronic disease, is the highest risk factor for global death, causing more than 10 million deaths every year (Collaborators, 2020). According to the latest survey results of global trends in hypertension prevalence, there were 1.28 billion hypertensive patients aged 30 to 79 in 2019, yet only about 20% of them had their blood pressure controlled (Collaboration, 2021). Furthermore, hypertension is frequently comorbid with glucose and lipid metabolism disorders, which markedly increases the risks of cardiovascular disease and death (Mottillo et al., 2010; Ji et al., 2013; Lin et al., 2021; Wang et al., 2021b). It is imperative to discover comprehensive and effective therapies to combat these disorders in this context.

Traditional Chinese medicine (TCM) is widely used to prevent and treat hypertension, dyslipidemia, diabetes, and other metabolic diseases in China (Yin et al., 2008; Wu et al., 2020). Fufang-Zhenzhu-Tiaozhi capsule (FTZ), a TCM patent prescription consisting of Rhizoma Coptidis, Radix Salivae Miltiorrhizae, Glossy Privet Fruit, Eucommia ulmoides Oliver, and four other herbs, has been widely used to treat glucolipid metabolic disorders in clinical practice for more than 20 years (Luo et al., 2018; Luo et al., 2019). Previous studies showed that FTZ exerted broad pharmacological effects, such as regulating lipid and glucose, anti-inflammation, anti-oxidation, improving insulin resistance, and protecting endothelial function (Chen et al., 2018; Bei et al., 2019; Song et al., 2021; Wang et al., 2021a). However, it is still unknown whether FTZ is effective for hypertension.

Recently, increasing evidence has suggested a critical role of gut microbiota in developing hypertension and its complications (Marques et al., 2018; Muralitharan et al., 2020). A notable decrease in intestinal microbial richness, diversity, and evenness has been observed in spontaneously hypertensive rats and chronic angiotensin II infusion rats (Yang et al., 2015). Consistent with animal studies, human studies have also found that the richness and diversity of intestinal microbiota in patients with pre-hypertension and hypertension were also significantly reduced as compared with the healthy subjects (Li et al., 2017). Furthermore, transplantation with the fecal microbiota from hypertensive patients into sterile mice led to elevated blood pressure (Li et al., 2017), implying that the abnormal intestinal flora plays a causal role in hypertension development.

Notably, both blood pressure and gut microbiota are profoundly influenced by dietary factors (Marques et al., 2018; Kolodziejczyk et al., 2019). Epidemiologic data show that increased fructose and salt intakes globally exceed the amount needed in most populations, substantially increasing the risks for hypertension and metabolic abnormalities (Brown et al., 2009; Kelishadi et al., 2014; Shen et al., 2019; Taskinen et al., 2019). Previous studies demonstrated that high-fructose and high-salt (HFS) diet-induced intestinal dysbiosis contributes to elevated blood pressure (Chen et al., 2020). In addition, the regulatory effects of FTZ on gut microbiota and its metabolites were reported (Luo et al., 2020; Shenghua et al., 2020).

This study aims to investigate whether FTZ treatment could alleviate HFS-induced hypertension and ascertain the role of intestinal microbiota in the antihypertensive effect of FTZ. Furthermore, the potential link between the regulation of intestinal microbiota and the hypotensive benefit of FTZ treatment is also to be discussed, which may contribute to a better understanding of the potential mechanisms of FTZ in the prevention and treatment of hypertension.

## 2 Materials and Methods

### 2.1 Drug Preparation

FTZ (Kangyuan Pharmaceutical Co., Ltd., Guangzhou, China) was extracted from eight herbs, including Rhizoma Coptidis (Huang Lian), Radix Salivae Miltiorrhizae (Dan Shen), Fructus Ligustri Lucidi (Nv Zhen Zi), Cortex Eucommiae (Du Zhong), Fructus Citri Sarcodactylis (Fo Shou), Rhizoma Atractylodis Macrocephalae (Bai Zhu), Radix Notoginseng (San Qi), and Herba Seu Radix Cirsii Japonici (Da Ji). The detailed preparation scheme, technical process, and storage method of FTZ capsules have been described in our previous study (Guo et al., 2011). Quality analysis of the FTZ extract was also performed *via* high-performance liquid chromatography (HPLC) fingerprinting as previously reported (Zhong et al., 2012).

### 2.2 Animals and Experimental Design

Male, specific pathogen-free (SPF) Wistar rats aged 5 weeks (Charles River Laboratory Animal Technology Company, Jiaxing, China) were caged and maintained in a temperature-controlled room with a 12-h light/dark cycle and free access to tap water and chow. Animal study procedures were approved by the Animal Studies Committee of Guangdong Pharmaceutical University.

#### 2.2.1 Experiment 1

**Figure 1:**
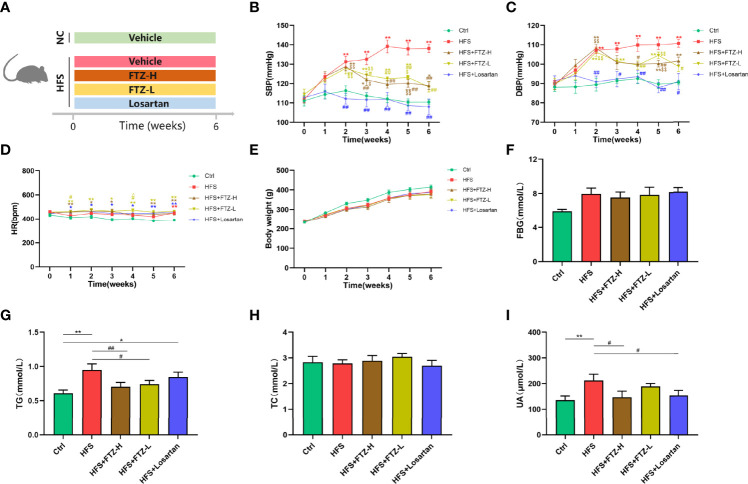
FTZ inhibited blood pressure elevation and improved lipid and uric acid metabolism in HFS-fed rats. **(A)** A schematic diagram for Experiment 1. **(B)** Systolic blood pressure. **(C)** Diastolic blood pressure. **(D)** Heart rate. **(E)** Body weight. **(F)** Fasting blood glucose. **(G–I)** Serum TG, TC, and UA. ^∗^ *p* < 0.05, ^∗∗^ *p* < 0.01 vs. Ctrl; ^#^ *p* < 0.05, ^##^ *p* < 0.01 vs. HFS ^$^ *p* < 0.05, ^$$^ *p* < 0.01 vs. HFS+Losartan. n = 8 per group. FTZ, Fufang-Zhenzhu-Tiaozhi capsule; HFS, high fructose and high salt; TG, triglyceride; TC, total cholesterol; UA, uric acid.

Rats were randomly divided into five groups: a control group (Ctrl) fed a normal chow (NC; Xietong Pharmaceutical Co., Ltd., Nanjing, China) and sterilized water, an HFS group (HFS) fed 20% fructose (Macklin Biochemical Co., Ltd., Shanghai, China) in drinking water and high salt diet (HSD; Xietong Pharmaceutical Co., Ltd., Nanjing, China), a high-dose FTZ-treated HFS group (HFS+FTZ-H) by gavage daily with 1.6 g/kg of FTZ, a low-dose FTZ-treated HFS group (HFS+FTZ-L) by gavage daily with 0.8 g/kg of FTZ, and a positive drug-treated HFS group (HFS+losartan) by gavage daily with 10 mg/kg of losartan (Merck Sharp & Dohme Pharmaceutical Co., Ltd., Hangzhou, China). NC (0.49% NaCI) and HSD (8% NaCI) were customized and identical in composition except for NaCl content. The body weight, blood pressure, and heart rate of all rats were recorded weekly. The feeding process lasted for 6 weeks. The schematic diagram for Experiment 1 is shown in **Figure 1A**. Blood samples and kidney tissues were collected and stored at −80°C for further usage.

#### 2.2.2 Experiment 2

This experiment comprised 3 phases with different interventions. In phase 1, rats were randomly divided into four groups: a control group (Ctrl) fed an NC and sterilized water, two HFS subgroups (HFS-A and HFS-B) fed 20% fructose in drinking water and 8% NaCl in the diet, and an FTZ-treated HFS group (HFS+FTZ) by gavage daily with 0.8 g/kg of FTZ (a more effective dosage according to the results in Experiment 1). This intervention process lasted for 6 weeks. In phase 2, with all the interventions in phase 1 unchanged, all rats were given an antibiotic cocktail daily by gavage feeding for 2 weeks. In phase 3, with the dietary intervention maintained, cross fecal microbiota transplantations (FMTs) were conducted for another 2 weeks between the Ctrl and HFS-A groups and the HFS-B and HFS-FTZ groups, respectively. The schematic diagram for Experiment 2 is shown in **Figure 4A**. The body weight, blood pressure, and heart rate of all the rats were recorded weekly, and the fresh fecal pellets were collected in the sixth, eighth, and tenth weeks.

### 2.3 Blood Pressure and Heart Rate Measurements

Systolic blood pressure (SBP), diastolic blood pressure (DBP), and heart rate (HR) of conscious rats were measured using the tail-cuff system (Softron BP-2010A, Beijing, China) as described previously (Yan et al., 2020). The measurements were performed during the daytime (13:00−18:00) in a quiet environment after a 3-day adaptive training. Each rat was measured five times, and the average value was used as the blood pressure and HR of the corresponding time point.

### 2.4 Enzyme Immunoassay

The serum contents of fasting blood glucose (FBG), triglyceride (TG), total cholesterol (TC), uric acid (UA), creatinine (Cr), and cystatin C (Cys-C) were detected by the commercially available enzyme immunoassay kits following the manufacturer’s instructions (Jiancheng Bioengineering Institute, Nanjing, China; Cloud-clone Corp., Wuhan, China).

### 2.5 Quantitative Reverse Transcriptase PCR

For quantitative reverse transcription PCR (qRT-PCR), total RNA isolation of kidney tissue was performed as previously described (Wang et al., 2017). Reverse transcription and SYBG qPCR were performed following the manufacturer’s instructions (Transgen Biotech, Beijing, China). Primers for AGT, AT1, TGF-β, α-SMA, TNF-α, IL-6, NOX-2, NOX-4, and β-Actin are listed in **Supplementary Table 1**. 2^−ΔΔCt^ method was applied to determine the relative changes in gene expression levels.

### 2.6 Histology

Masson staining was used to visualize histological morphology and fibrosis in kidney tissue. Before each staining, kidney tissue was fixed in 4% paraformaldehyde solution for 1 week and embedded in paraffin. Transverse sections that are 4 µm thick were cut and then stained with a Masson staining kit (Solarbio Science & Technology Co., Ltd., Beijing, China) following the manufacturer’s instructions. All stained sections were examined using light microscopy (×100 and ×200 magnification). The positive area was analyzed using Image-Pro Plus software.

### 2.7 Fecal Microbiota Transplantation

The FMT materials were obtained from the rats in the sixth week of Experiment 2. The rats were stimulated to defecate by gentle means, such as grasping and rectal massage. The fecal pellets were collected directly in a sterile cryotube, quick-frozen in liquid nitrogen immediately, and subsequently stored at −80°C until use. The recipient rats were then given an antibiotic cocktail by gavage daily for 2 weeks until 48 h before FMT. The antibiotic cocktail comprised ampicillin (MedChemExpress, Monmouth Junction, NJ, USA; 110 mg/kg), vancomycin (MedChemExpress; 55 mg/kg), metronidazole (MedChemExpress; 110 mg/kg), and neomycin (MedChemExpress; 110 mg/kg) as previously reported (Yan et al., 2020). The gavage volume was 2.5 ml/kg, and the antibiotic cocktail solution was thoroughly mixed before each gavage. The fecal pellets collected in phase 1 were added with sterile phosphate-buffered saline (PBS) buffer (10 ml/g, containing 20% sterile glycerol). The fecal samples of each group were fully mixed and resuspended. After the fecal mixture suspension solution was centrifugated at 2,000 rpm at 4°C for 10 min, the supernatants were collected and quickly stored at −80°C until use. The above operations were conducted under sterile conditions. The recipient rats were orally inoculated with 1 ml of prepared fecal contents from donor rats once a day.

### 2.8 16S rRNA Gene Sequencing

Total genomic DNA from fecal samples in the sixth, eighth, and tenth weeks was extracted using cetyl trimethylammonium bromide (CTAB) or sodium dodecyl sulfate (SDS) method. DNA concentration was determined by NanoDrop. The purity and integrity could be evaluated using 1% agarose gel electrophoresis. DNA was diluted to 1 ng/μl using sterile water according to the concentration. 16S rRNA genes were amplified using the specific primer with the barcode. The same volume of 1× loading buffer was mixed (contained SYBR Green) with PCR products, and electrophoresis was operated on 2% agarose gel for detection. Samples with a bright main strip between 400 and 450 bp were chosen for further experiments. Amplicons were pooled in equal proportions and purified using TIANgel Purification Kit (TIANGEN Biotech, Beijing, China). The purified product was used to prepare the Illumina DNA library. Sequencing libraries were generated using TIANSeq Fast DNA Library Prep Kit (Illumina) (TIANGEN Biotech). The library quality was assessed on the Qubit@ 2.0 Fluorometer (Thermo Scientific, Waltham, MA, USA) and Agilent Bioanalyzer 2100 system. Finally, the library was sequenced on the Illumina platform using the 2 × 250 bp paired-end protocol. According to the official tutorials, microbiome bioinformatics was performed with QIIME 2, with slight modifications. Briefly, raw sequence data were demultiplexed using the demux plugin, followed by primer cutting with the cutadapt plugin. Sequences were then quality filtered, denoised, and merged, and chimera was removed using the DADA2 plugin. Species annotation was performed using QIIME2 software. For 16S, the annotation database is Silva Database. Multiple sequence alignment was performed using QIIME2 software to study the phylogenetic relationship of each amplicon sequence variant (ASV) and the differences of the dominant species among different samples (groups). Chao1 and Shannon indexes were used to quantify and compare microbial species richness and alpha diversity. Principal coordinate analysis (PCoA) based on unweighted unifrac distances was performed to obtain principal coordinates and visualize differences of samples in complex multi-dimensional data. Bacterial taxa within different groups were arranged based on their relative abundance (false discovery rate <0.05). PICRUSt2 was used to predict the pathway abundances, and STAMP software confirmed the groups’ differences.

### 2.9 Statistical Analysis

Data were presented as mean ± SEM or median (minimum to maximum). The Shapiro–Wilk test was used to evaluate data normality. For normally distributed data, one-way ANOVA and *post-hoc* test (least significant difference) were used for multi-group comparison. The Kruskal–Wallis test was used for multi-group comparison for data not distributed normally. Statistical analysis was performed using SPSS v25 (IBM, Armonk, NY, USA). The *p* < 0.05 was considered statistically significant.

## 3 Results

### 3.1 Fufang-Zhenzhu-Tiaozhi Capsule Inhibited Blood Pressure Elevation in High-Fructose and High-Salt Diet-Fed Rats

To evaluate the effect of FTZ on blood pressure, rats fed with HFS were treated with a high or low dose of FTZ, losartan, or vehicle by gavage for 6 weeks. Following the initiation of drug administration, the SBP and DBP of HFS-fed rats were gradually elevated and significantly higher than those of NC-fed rats at the end of the second week, subsequently reaching a plateau from the fifth week (**Figures 1B, C**). However, when the HFS-fed rats were administrated with a high or low dose of FTZ simultaneously, the blood pressure-increasing effect of the HFS diet was suppressed from the third week. Then the blood pressure of FTZ-treated HFS rats remained significantly lower than that of untreated HFS rats (**Figures 1B, C**). Additionally, the SBP and DBP of losartan-treated rats were similar to those of NC-fed rats (**Figures 1B, C**). Notably, the HR of all HFS-fed rats showed no apparent difference between groups but was higher than that of NC-fed rats (**Figure 1D**). These data reveal that FTZ administration alleviates HFS-induced hypertension.

### 3.2 Fufang-Zhenzhu-Tiaozhi Capsule Improved Metabolic Disorders of Lipid and Uric Acid in High-Fructose and High-Salt Diet-Fed Rats

The effects of FTZ on metabolism were assessed by measuring glucose, lipid, and UA parameters and body weight. After 6-week HFS feeding, serum TG and UA concentrations significantly increased, while FTZ treatment, especially with a high dosage, markedly improved these disorders (**Figures 1G, I**). Additionally, all HFS-fed rats showed slightly higher FBG levels and lower body weight than NC-fed rats, while FTZ treatment made no significant difference (**Figures 1E, F**). Losartan treatment decreased the level of serum UA but did not impact body weight and serum TG and FBG levels (**Figures 1E–G, I**). Moreover, no significant difference in TC was observed between all groups (**Figure 1H**). These results imply that FTZ administration alleviates HFS-induced lipid and UA metabolism disorders.

### 3.3 Fufang-Zhenzhu-Tiaozhi Capsule Reduced Renal Fibrosis in High-Fructose and High-Salt Diet-Fed Rats

**Figure 2:**
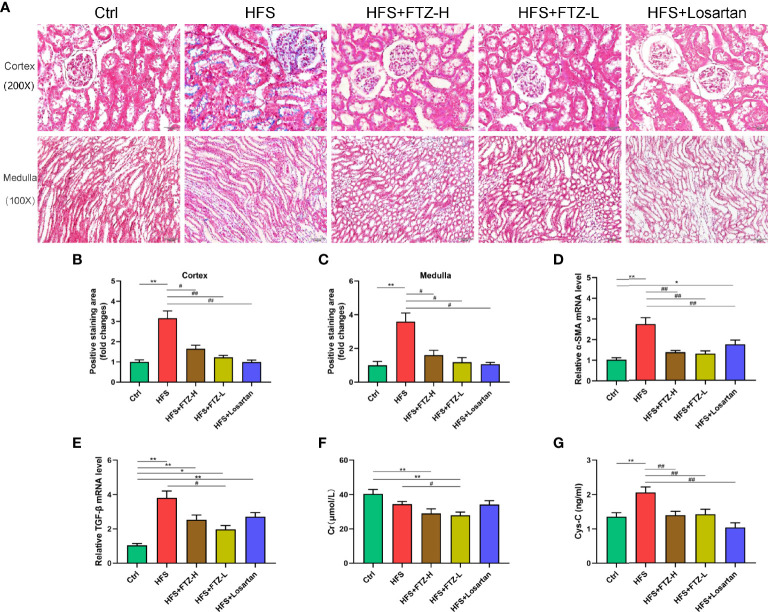
FTZ reduced renal fibrosis and improved renal function in HFS-fed rats. **(A)** Masson staining of kidney sections. **(B)** Positive staining area in renal cortex. **(C)** Positive staining area in renal medulla. **(D, E)** Relative mRNA expression of α-SMA and TGF-β in renal cortex. **(F, G)** Serum Cr and Cys-C. ^∗^ *p* < 0.05, ^∗∗^ *p* < 0.01 vs. Ctrl; ^#^ *p* < 0.05, ^##^ *p* < 0.01 vs. HFS. n = 8 per group. FTZ, Fufang-Zhenzhu-Tiaozhi capsule; HFS, high fructose and high salt; Cr, creatinine; Cys-C, cystatin C.

The serum concentrations of Cr and Cys-C were measured, two indicators of kidney function. Intriguingly, the serum Cr level in HFS-fed rats was not higher than in NC-fed rats (**Figure 2F**), presumably because the serum Cr level is only elevated when the kidney is severely damaged. Despite this, we found a lower level of serum Cr in FTZ-treated HFS-fed rats than in HFS-fed rats (**Figure 2F**). Notably, the serum level of Cys-C, a more accurate and sensitive biomarker for early renal injury, was significantly elevated in HFS-fed rats and reduced by FTZ treatment (**Figure 2G**). These data were further supported by Masson staining. The results demonstrated that the positive area of fibrosis in HFS-fed rats was markedly increased compared with NC-fed rats. At the same time, FTZ administration effectively inhibited the extension of the fibrotic area caused by the HFS diet (**Figures 2A–C).** The mRNA levels of α-SMA and TGF-β in renal cortical tissues were upregulated by the HFS diet and declined during FTZ treatment (**Figures 2D, E**). These data collectively infer that FTZ attenuates HFS-induced renal fibrosis.

### 3.4 Fufang-Zhenzhu-Tiaozhi Capsule Alleviated Renal Gene Expression of Inflammation Cytokines, NADPH Oxidases, and Renin–Angiotensin System in High-Fructose and High-Salt Diet-Fed Rats

**Figure 3:**
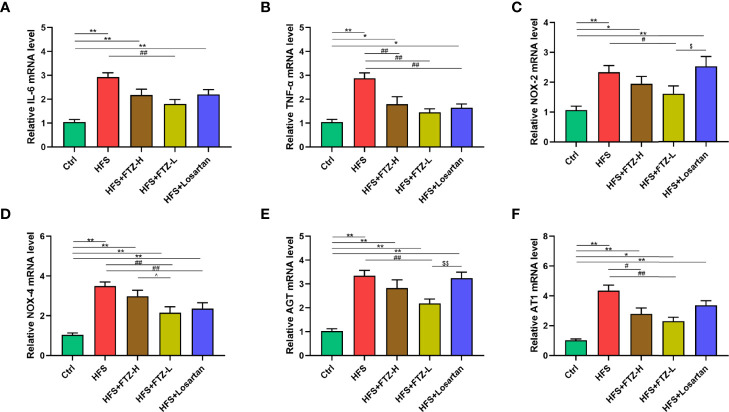
FTZ alleviated renal gene expression of inflammation cytokines, NADPH oxidases, and RAS in HFS-fed rats. **(A, B)** Relative mRNA expression of IL-6 and TNF-α in renal cortex. **(C, D)** Relative mRNA expression of NOX-2 and NOX-4 in renal cortex. **(E, F)** Relative mRNA expression of AGT and AT1 in renal cortex. ^∗^ *p* < 0.05, ^∗∗^ *p* < 0.01 vs. Ctrl; ^#^ *p* < 0.05, ^##^ *p* < 0.01 vs. HFS; ^^^ *p* < 0.05 vs. HFS+HFZ-H; ^$^ *p* < 0.05, ^$$^ *p* < 0.01 vs. HFS+losartan. n = 8 per group. FTZ, Fufang-Zhenzhu-Tiaozhi capsule; RAS, renin–angiotensin system; HFS, high fructose and high salt.

Hypertension and renal fibrosis are associated with inflammation and oxidative stress. NADPH oxidases are the enzymes that produce reactive oxygen species as primary products, which are closely associated with oxidative stress. To determine whether FTZ intervention could attenuate the renal inflammation and oxidative stress triggered by the HFS diet, mRNA expression levels of the representative indicators of inflammatory cytokines (IL-6 and TNF-α) and NADPH oxidases (NOX-2 and NOX-4) were analyzed in renal cortical tissues. The results revealed that the highly expressed mRNA levels of TNF-α, IL-6, NOX-2, and NOX-4 were downregulated by FTZ treatment (**Figures 3A–D**), suggesting the potential favorable effects of FTZ on HFS-induced renal inflammation and oxidative stress.

The renin–angiotensin system (RAS) plays an essential role in HFS-induced hypertension. Previous studies showed that the HFS diet elevated the renin and Ang II levels in both serum and urine and the mRNA level of AGT in renal cortical tissues. In contrast, vancomycin treatment lowered the increased blood pressure and suppressed the intrarenal but not systemic RAS (Chen et al., 2020). This study examines whether FTZ treatment could reduce the mRNA levels of RAS components in renal cortical tissues. Notably, the increased renal mRNA expression levels of AGT and AT1 were downregulated by FTZ treatment (**Figures 3E, F**). Consequently, these results indicate that the blood pressure-lowering effect of FTZ is probably due to the inactivation of intrarenal RAS.

### 3.5 Fufang-Zhenzhu-Tiaozhi Capsule Restored Gut Dysbiosis in High-Fructose and High-Salt Diet-Fed Rats

**Figure 4:**
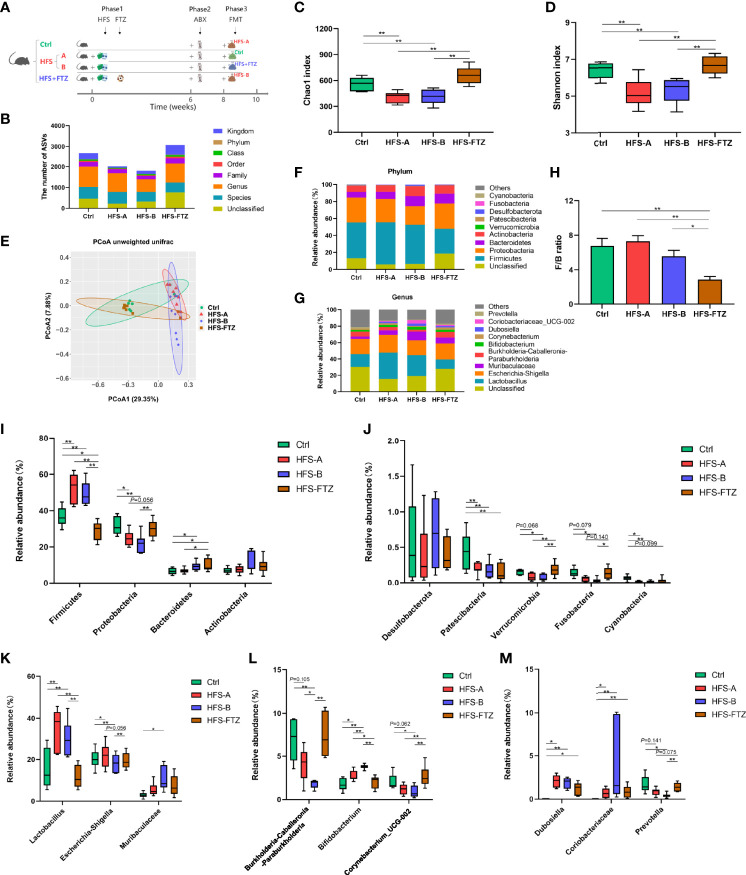
Significant changes of gut microbiota in response to FTZ treatment. **(A)** A schematic diagram for Experiment 2. **(B)** ASV number. **(C)** Chao1 index. **(D)** Shannon index. **(E)** β-Diversity based on unweighted unifrac PCoA. **(F)** Relative abundance of microbiota compositions at the phylum level. **(G)** Relative abundance of microbiota compositions at the genus level. **(H)** Ratio of Firmicutes to Bacteroidetes. **(I, J)** Relative abundances of the top 9 bacterial phyla, including Firmicutes, Proteobacteria, Bacteroidetes, Actinobacteria, Desulfobacterota, Patescibacteria, Verrucomicrobia, Fusobacteria, and Cyanobacteria. **(K–M)** Relative abundances of the top 9 bacterial genera, including *Lactobacillus*, *Escherichia-Shigella*, Muribaculaceae, *Burkholderia-Caballeronia-Paraburkholderia*, *Bifidobacterium*, *Corynebacterium*, *Dubosiella*, *Coriobacteriaceae_UCG-002*, and *Prevotella*. ^∗^ *p* < 0.05, ^∗∗^ *p* < 0.01. n = 6~8 per group. ABX, antibiotics; FMT, fecal microbiota transplantation. FTZ, Fufang-Zhenzhu-Tiaozhi capsule; ASV, amplicon sequence variant; PCoA, principal coordinate analysis.

Previous studies showed that gut dysbiosis was associated with the activation of intrarenal RAS, which mediates HFS-induced hypertension (Chen et al., 2020). Experiment 2 **(** **Figure 4A** **)** was undertaken to examine and verify the regulatory effect of FTZ on gut microbiota by sequencing the bacterial 16S rRNA V3–V4 region in feces, which suggests the potential mechanisms of FTZ’s therapeutic effects on hypertension. Almost consistent with previous studies, after a 6-week intervention in phase 1, there were a lower number of ASVs and less intestinal microbial richness and diversity in HFS-fed rats than in NC-fed rats, as evidenced by the two decreased alpha diversity indexes, Chao1 and Shannon (**Figures 4B–D**). Beta diversity of intestinal microbiota evaluated by unweighted unifrac-based PCoA also showed that HFS-fed rats exhibited a substantially different microbiota composition than NC-fed rats (**Figure 4E**). On the contrary, FTZ-treated HFS-fed rats displayed a remarkably similar gut microbiota structure to NC-fed rats (**Figure 4E**). The microbial richness and diversity and ASVs in FTZ-treated HFS-fed rats were also strikingly improved compared to those in HFS-fed rats (**Figures 4B–D**).

Overall microbial compositions at different levels were further analyzed (**Figures 4F, G**). Phylum-level analysis showed that HFS feeding significantly reduced the relative abundance of Proteobacteria, Patescibacteria, Verrucomicrobia, Fusobacteria, and Cyanobacteria and a significant increase in Firmicutes (**Figures 4I–J**). However, when HFS-fed rats were treated with FTZ, there was a significant increase in the relative abundance of Proteobacteria, Verrucomicrobia, and Fusobacteria, and a significant decrease in Firmicutes and the F/B ratio (**Figures 4H–J**). Further analysis at the genus level revealed that HFS-fed rats had a higher relative abundance of *Lactobacillus*, *Bifidobacterium*, *Dubosiella*, and *Coriobacteriaceae_UCG-002* and a lower abundance of *Burkholderia-Caballeronia-Paraburkholderia*, *Corynebacterium*, and *Prevotella* compared with NC-fed rats (**Figures 4K–M**). However, when HFS-fed rats were administered FTZ, a significant decrease in the relative abundance of *Lactobacillus* and *Bifidobacterium* and a significant increase in that of *Burkholderia-Caballeronia-Paraburkholderia*, *Corynebacterium*, and *Prevotella* were observed (**Figures 4K–M**).

**Figure 5:**
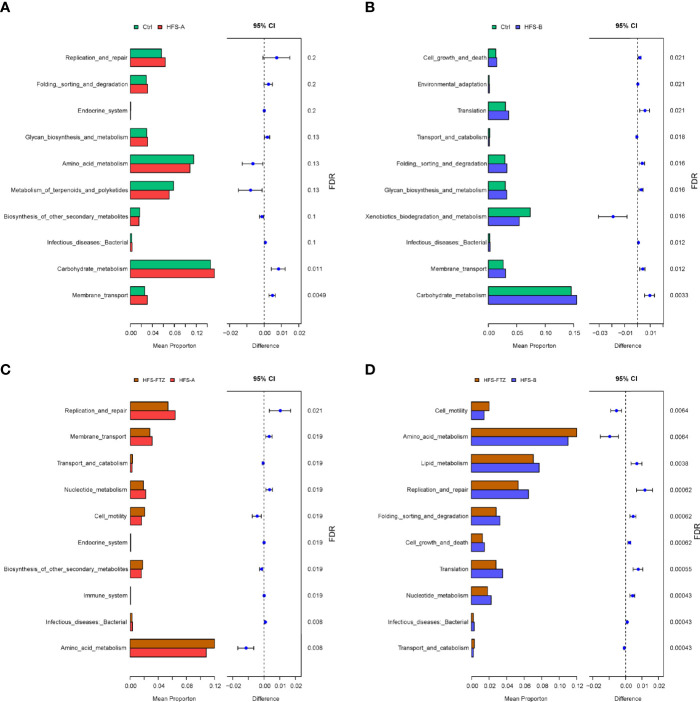
KEGG metabolic pathway analysis of differential functional genes. **(A)** Functional differences between the Ctrl and HFS-A groups. **(B)** Functional differences between the Ctrl and HFS-B groups. **(C)** Functional differences between the HFS-FTZ and HFS-A groups. **(D)** Functional differences between the HFS-FTZ and HFS-B groups. KEGG, Kyoto Encyclopedia of Genes and Genomes; HFS, high fructose and high salt.

Difference analysis of Kyoto Encyclopedia of Genes and Genomes (KEGG) metabolic pathways using PICRUSt2 was performed to find the abundance differences of functional genes within the microbial community between groups. The results showed that the genes related to carbohydrate metabolism were upregulated significantly in HFS-fed rats compared to those in NC-fed rats (**Figures 5A–D**). Furthermore, FTZ treatment upregulated the genes related to amino acid metabolism but downregulated the genes involved in lipid metabolism and replication and repair. These data, in combination, indicate that FTZ treatment regulates HFS-induced gut dysbiosis.

### 3.6 Transplantation With Fufang-Zhenzhu-Tiaozhi Capsule-Modulated Gut Microbiota Decreased the Blood Pressure in High-Fructose and High-Salt Diet-Fed Rats

**Figure 6:**
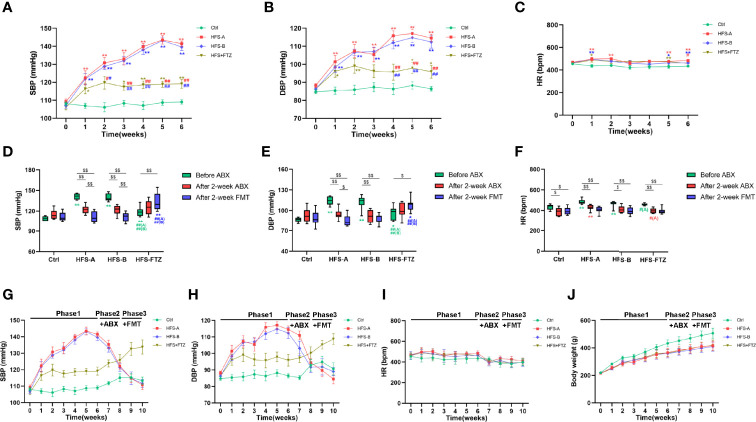
Transplantation with FTZ-modulated gut microbiota decreased the blood pressure in HFS-fed rats. **(A)** SBP before ABX intervention. **(B)** DBP before ABX intervention. **(C)** HR before ABX intervention. **(D)** SBP after ABX and FMT interventions. **(E)** DBP after ABX and FMT interventions. **(F)** HR after ABX and FMT interventions. **(G)** SBP in these three phases. **(H)** DBP in these three phases. **(I)** HR in these three phases. **(J)** Body weight in these three phases. ^∗^ *p* < 0.05, ^∗∗^ *p* < 0.01 vs. Ctrl; ^#^ *p* < 0.05, ^##^ *p* < 0.01 vs. HFS-A/B; ^$^ *p* < 0.05, ^$$^ *p* < 0.01. n = 8 per group. FTZ, Fufang-Zhenzhu-Tiaozhi capsule; HFS, high fructose and high salt; SBP, systolic blood pressure; ABX, antibiotics; DBP, diastolic blood pressure; HR, heart rate; FMT, fecal microbiota transplantation.

There is growing evidence from animal and clinical studies suggesting that FMT effectively treats hypertension (Kim et al., 2018; Zhong et al., 2021). Hence, FMT was performed to determine whether FTZ exerted the blood pressure-lowering effect by modulating gut microbiota. After a 6-week intervention in phase 1, the results demonstrated similar trends and differences in SBP, DBP, and HR between the Ctrl group, HFS group, and FTZ group in Experiment 1 (**Figures 1B–D**, **Figures 6A–C**). After a 2-week antibiotic intervention, the HRs in all groups were decreased in phase 2 and remained at a low level during the FMT period in phase 3 (**Figures 6F, I**). Additionally, in phases 2 and 3, the body weights in all groups had the same growth trends and differences as those in phase 1, probably suggesting no ill effects due to either antibiotics or FMT treatment (**Figure 6J**).

More importantly, the elevated SBP and DBP of HFS-fed rats were significantly decreased after antibiotic treatment and further decreased to near-normal levels after transplantation with intestinal flora from NC-fed rats or FTZ-treated HFS-fed rats (**Figures 6D, E, G, H**). In contrast, the alleviated SBP and DBP of FTZ-treated HFS-fed rats were gradually elevated after antibiotic treatment and further increased after transplantation with intestinal flora from HFS-fed hypertensive rats (**Figures 6D, E, G, H**). No significant changes in SBP and DBP were observed in NC-fed rats (**Figures 6D, E, G, H**). These data indicate that the therapeutic effects of FTZ on HFS-induced hypertension could be transferred through FMT.

Meanwhile, the results also demonstrated that transplantation with normal intestinal flora shaped by a regular diet could abrogate the blood pressure-elevating effect of the HFS diet, and transplantation with disordered intestinal flora induced by the HFS diet could hardly elevate the blood pressure in the context of normal diet (**Figures 6D, E, G, H**). Thus, these data also imply that the HFS diet and its induced gut dysbiosis are interdependent in contributing to the development of hypertension.

### 3.7 Transplantation With Fufang-Zhenzhu-Tiaozhi Capsule-Modulated Intestinal Microbiota Reshaped Gut Flora in High-Fructose and High-Salt Diet-Fed Rats

**Figure 7:**
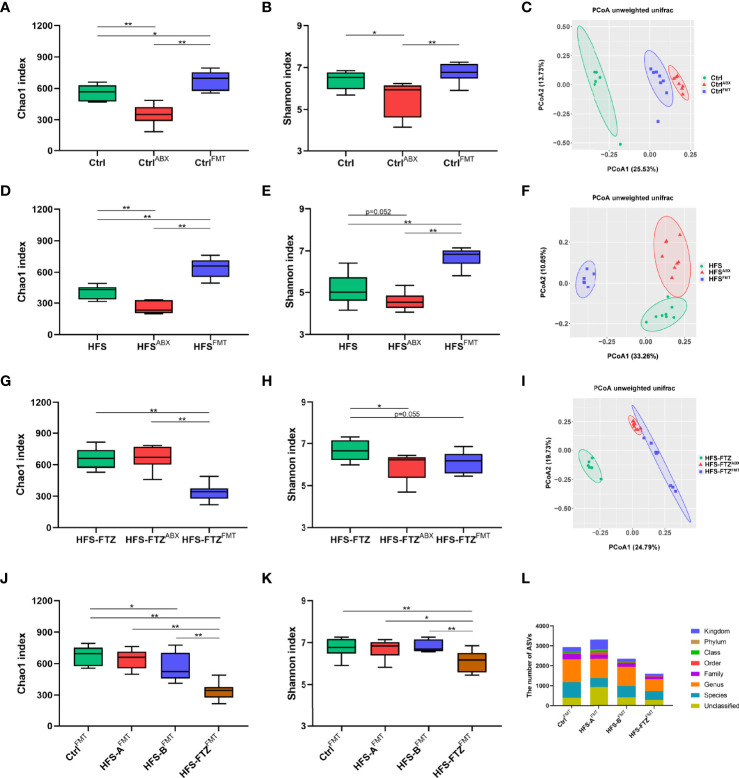
Transplantation with FTZ-modulated intestinal microbiota reshaped gut flora in HFS-fed rats. **(A–C)** Significant changes in Ctrl group’s gut microbiota after ABX and FMT interventions. **(D–F)** Significant changes in HFS group’s gut microbiota composition after ABX and FMT interventions. **(G–I)** Significant changes in HFS-FTZ group’s gut microbiota composition after ABX and FMT interventions. **(J)** Chao1 index after FMT intervention. **(K)** Shannon index after FMT intervention. **(L)** ASV number after FMT intervention. ^∗^ *p* < 0.05, ^∗∗^ *p* < 0.01. n = 6~8 per group. Ctrl^ABX^, Ctrl group after ABX intervention; Ctrl^FMT^, Ctrl group after FMT intervention; HFS^ABX^, HFS group after ABX intervention; HFS^FMT^, HFS group after FMT intervention; HFS-FTZ^ABX^, HFS-FTZ group after ABX intervention; HFS-FTZ^FMT^, HFS-FTZ group after FMT intervention. FTZ, Fufang-Zhenzhu-Tiaozhi capsule; HFS, high fructose and high salt; ABX, antibiotics; FMT, fecal microbiota transplantation; ASV, amplicon sequence variant.

To obtain more robust evidence, the gut microbiota profiles of the rat feces were analyzed after antibiotic and FMT interventions. Beta diversity analysis revealed that each group exhibited distinctly different gut microbiota compositions in the three phases with various treatments, suggesting the effectiveness of antibiotics and FMT interventions (**Figures 7C, F, I**). Additionally, alpha diversity analysis indicated that the decreased microbial richness and diversity induced by antibiotic treatment in NC-fed and HFS-fed rats were remarkably improved after FMT (**Figures 7A, B, D, E**). Notably, antibiotic treatment only reduced the diversity of intestinal flora in FTZ-treated HFS-fed rats but had no apparent effect on intestinal microbial richness (**Figures 7G, H**). These results imply a potential protective role of FTZ in preventing the reduction of intestinal microbial richness caused by antibiotics. Moreover, transplantation with intestinal flora from HFS-fed donor rats significantly reduced the intestinal flora richness of FTZ-treated HFS-fed receiver rats. However, it had no significant effect on diversity. In summary, these data suggest that FMT reconstructs intestinal microbiota compositions in receiver rats.

### 3.8 Reshaped Intestinal Microbiota by Transplantation With Fufang-Zhenzhu-Tiaozhi Capsule-Modulated Gut Flora Contributed to the Hypotensive Effect

The gut microbiota profiles in all groups after FMT intervention were examined to clarify the causal relationship between the reshaped intestinal microbiota and the decreased blood pressure. Consistent with the well-known conclusion that high blood pressure is associated with low richness and diversity of gut flora, the lowest intestinal microbial richness, diversity, and ASVs number occurred in FTZ-treated HFS-fed rats after FMT treatment, which was concurrently accompanied by the highest blood pressure (**Figures 6D, E**, **Figures 7J–L**). In addition, after crossing FMT to each other, no significant differences in alpha diversity and blood pressure were observed between the NC-fed and HFS-fed rats (**Figures 6D, E**, **Figures 7J–L**). Such outcomes may indicate that during FMT, NC diet and transplantation with NC diet-shaped normal gut flora are more predominant in gut flora reconstruction than transplantation with HFS-induced abnormal intestinal microbiota and HFS diet, respectively.

**Figure 8:**
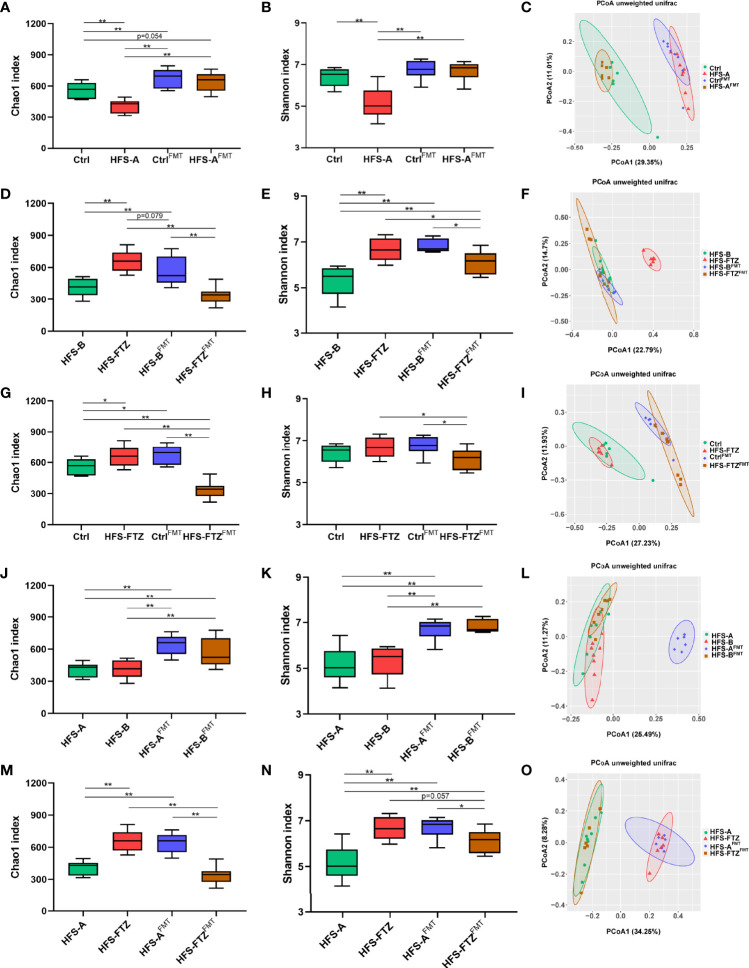
Reshaped intestinal microbiota by transplantation with FTZ-modulated gut flora contributed to the hypotensive effect. **(A–C)** Comparative analysis of gut microbiota between the Ctrl and HFS-A groups. **(D–F)** Comparative analysis of gut microbiota between the HFS-B and HFS-FTZ groups. **(G–I)** Comparative analysis of gut microbiota between the Ctrl and HFS-FTZ groups. **(J–L)** Comparative analysis of gut microbiota between the HFS-A and HFS-B groups. **(M–O)** Comparative analysis of gut microbiota between the HFS-A and HFS-FTZ groups. ^∗^ *p* < 0.05, ^∗∗^ *p* < 0.01. n = 6~8 per group. FTZ, Fufang-Zhenzhu-Tiaozhi capsule; HFS, high fructose and high salt.

A comprehensive analysis of the gut microbiota profiles before and after cross FMT was undertaken to elucidate further the relationship between blood pressure, diet, and gut flora. The results of beta diversity analysis showed that the gut microbiota compositions of most cross FMT receiver rats were remarkably similar to those of the cross FMT donor rats (**Figures 8C, F**). Intriguingly, transplantation with intestinal flora from FTZ-treated HFS-fed donor rats failed to make HFS-fed receiver rats such a shift in gut microbiota composition (**Figure 8F**). Such data infer that the HFS diet has a more dominant role in microbiota-shaping effects than transplantation with FTZ-modulated gut microbiota during FMT.

In terms of alpha diversity, both NC-fed rats’ and HFS-fed rats’ decreased intestinal microbial richness and diversity induced by antibiotic treatment were remarkably improved after cross FMT (**Figures 7A, B, D, E**). The differences in richness and diversity between these two groups were also significantly diminished after cross FMT (**Figures 8A, B**). Meanwhile, the blood pressure of these two groups was at similarly low levels (**Figures 6D, E**). These results suggest the potential therapeutic effects of the NC diet and NC diet-shaped normal flora against the blood pressure-elevating effects of HFS-induced intestinal dysbiosis and HFS diet, respectively.

On the contrary, the intestinal microbial richness and diversity of HFS-fed and FTZ-treated HFS-fed rats were reversed after antibiotics and cross FMT (**Figures 8D, E**). This could be primarily attributed to the regulatory effect of FMT, considering that these two groups were in the same context of the HFS diet. Interestingly, these two groups simultaneously presented with opposite blood pressure trends (**Figures 6D, E**). Therefore, these results indicate that FTZ-regulated gut flora, as with the normal gut flora, could effectively suppress the blood pressure-elevating effect of the HFS diet.

To study the effects of diet and transplanted flora on blood pressure, the blood pressure and intestinal flora of NC-fed rats and FTZ-treated HFS-fed rats were compared. Before antibiotic and FMT treatments, the blood pressure of these two groups was significantly lower than that of the two HFS subgroups (**Figures 6D, E** **)**. Their intestinal microbial richness and diversity were also at high levels (**Figures 4C, D**, **Figures 8G, H**). Moreover, beta diversity analysis showed similar compositions of these two groups, which differed from those of the two HFS subgroups (**Figure 4E**, **Figure 8I**). After transplantation with the dysregulated intestinal flora induced by the HFS diet, these two groups’ blood pressure and gut flora profiles showed inconsistent changes due to their different diets (**Figures 6D, E**, **Figures 8G–I**). These data imply that maintaining an HFS diet is necessary for the blood pressure-increasing effect of HFS-disturbed flora. In contrast, keeping a normal diet could resist the transplanted abnormal flora and its blood pressure-increasing effect.

The blood pressure and gut microbiota profiles of the two HFS subgroups were compared to clarify further the effects of diet and transplanted flora on blood pressure. Before antibiotic and FMT treatments, no apparent differences in blood pressure and gut microbial richness and diversity existed in these two HFS subgroups (**Figures 6D, E**, **Figures 8J, K**). Moreover, after being transplanted with NC-shaped normal flora and FTZ-remodeled flora, these two HFS subgroups still demonstrated similar blood pressure levels and microbial richness and diversity (**Figures 6D, E**, **Figures 8J, K**). Interestingly, beta diversity analysis showed different gut microbiota compositions in these two groups (**Figure 8L**). This may be because FMT increased the minor differences between these two HFS subgroups. Nevertheless, these results indicate that both the normal gut flora and the FTZ-reshaped intestinal flora could inhibit HFS-induced high blood pressure.

Finally, the blood pressure and intestinal flora profiles of the FTZ-treated and untreated HFS-fed groups were compared. Before antibiotic and FMT interventions, the blood pressure of FTZ-treated HFS-fed rats was significantly lower than that of HFS-fed rats (**Figures 6D, E**), and the intestinal microbial richness and diversity of FTZ-treated HFS-fed rats were markedly higher compared with HFS-fed rats (**Figures 4C, D**, **Figures 8M, N**). Beta diversity analysis also showed distinctly different gut microbiota compositions between these two groups (**Figure 4E**, **Figure 8O**). After being transplanted with HFS-induced abnormal gut flora and NC-shaped normal gut flora, the blood pressure of FTZ-treated HFS-fed receiver rats increased further **(** **Figures 6D, E** **)**. In contrast, the blood pressure of HFS-fed receiver rats decreased further (**Figures 6D, E**). In addition, the intestinal microbial richness and diversity of these two groups appeared to be exchanged (**Figures 8M, N**). Beta diversity analysis also showed different intestinal microbial compositions of these two groups (**Figure 8O**). Moreover, HFS-fed receiver rats demonstrated a similar gut flora composition to FTZ-treated HFS-fed rats (**Figure 8O**). FTZ-treated HFS-fed receiver rats displayed a similar gut flora composition to NC-fed rats (**Figure 8O**). These results indirectly indicate the similarities of gut microbiota compositions between FTZ-treated HFS-fed and NC-fed rats and between the HFS-fed rats in these two subgroups. Furthermore, these results suggest that when maintaining an HFS diet, transplantation with HFS-disturbed gut flora can promote an increase in blood pressure. In contrast, transplantation with normal flora can inhibit the increase of blood pressure.

## 4 Discussion

Hypertension is highly prevalent worldwide. Although there are many antihypertensive drugs, the control rate of hypertension is still low. Additionally, hypertension is frequently comorbid with metabolic disorders, and these diseases interact closely with each other, which makes the treatment more difficult. Hence, it is urgent to develop novel, effective, and safe therapeutic agents.

In our diet-induced hypertension model, HFS-fed rats exhibited markedly elevated blood pressure and serum levels of TG and UA, whereas FTZ administration significantly suppressed these increasing effects. HFS diet has a complex synergistic effect on promoting the increase of blood pressure. Excessive dietary fructose intake contributes to the development of sodium-induced hypertension through multiple mechanisms (Eren et al., 2019). In addition, fructose was reported to induce hypertriglyceridemia and hyperuricemia (Nakagawa et al., 2006). Previous studies showed an elevated level of serum TG in HFS-induced hypertensive rats (Chen et al., 2020). Notably, an epidemiological study revealed that serum TG level was considerably associated with the development of hypertension (Tomita et al., 2021). Hyperuricemia was also involved in the development of hypertension since it activated RAS and inhibited nitric oxide synthesis (Ponticelli et al., 2020). Additionally, a clinical study found that allopurinol lowered the serum UA level and prevented blood pressure elevation caused by excessive fructose intake (Perez-Pozo et al., 2010).

Renal mechanisms, including the activation of intrarenal RAS and sympathetic nerves, the decreased renal nitric oxide production, and the increased renal UA and reactive oxygen species production and sodium retention, play a crucial role in HFS-induced hypertension (Xu and Yang, 2018; Komnenov et al., 2019). In this study, Masson staining of kidney tissues revealed significant renal fibrosis in HFS-fed rats. The mRNA expression levels of inflammatory cytokines and NADPH oxidases, and biomarkers of fibrosis and RAS components in renal cortical tissues were also upregulated. However, these abnormalities were ameliorated after FTZ administration. Thus, these results imply that FTZ exhibits beneficial efficacy against HFS-induced hypertension, related metabolic disorders, and renal injury, which may be related to the inhibition of intrarenal RAS activation, inflammatory reaction, and oxidative stress.

Previous work had suggested that the intestinal microbial imbalance was related to renal RAS activation, which regulated HFS-induced hypertension (Chen et al., 2020). Notably, there is accumulating evidence supporting that gut dysbiosis is linked to the development of hypertension (Yang et al., 2015). Intestinal flora can regulate blood pressure directly by affecting the production of vasoactive hormones (such as dopamine, norepinephrine, and serotonin) (Afsar et al., 2016). Moreover, intestinal microbial metabolites, such as short-chain fatty acids, also mediate systemic or local RAS to regulate blood pressure. A previous study showed that sodium butyrate inhibited angiotensin II-induced hypertension by inhibiting renal (pro)renin receptor and intrarenal RAS (Wang et al., 2017). Acetate supplementation was also reported to result in the downregulation of RAS in the kidney (Marques et al., 2017). Moreover, propionate regulated renin release from afferent arterioles in an olfr78 receptor-dependent manner (Pluznick, 2014). TMAO, a carnitine-derived intestinal microbial metabolite, also prolonged the blood pressure-raising effect of angiotensin II (Ufnal et al., 2014). In addition, uremic toxins (indophenol sulfate and *p*-cresol sulfate) derived from intestinal flora caused chronic renal injury by activating intrarenal RAS (Sun et al., 2012).

Therefore, restoring intestinal dysbiosis is considered a promising therapy in hypertension management. Many natural herbal products effectively manage metabolic syndrome by modifying gut microbiota (Shabbir et al., 2021). The herbal formulas of TCM, consisting of several herbal medicines, have also shown blood pressure-lowering and gut flora-modifying effects. Zhengganxifeng decoction regulated intestinal flora and decreased blood pressure *via* mediating RAS in spontaneously hypertensive rats (Yu et al., 2019). The combination of *E. ulmoides* and *Tribulus terrestris* also exerted an antihypertensive effect in spontaneously hypertensive rats, which was associated with the modulation of intestinal microbiota and their beneficial metabolites (Qi et al., 2020). In addition, Xiaoqinglong decoction reduced the blood pressure of Dahl salt-sensitive rats and improved intestinal microbiota composition, and transplantation with fecal microbiota treated by Xiaoqinglong decoction also achieved similar results in hypotensive effects (Zhou et al., 2019).

FTZ is a widely used TCM patent prescription consisting of many chemical substances, from which 44 substances have been identified (Zhong et al., 2012). Many of these substances, including Berberine (Xu et al., 2020), Salidroside (Li et al., 2020), Ginsenoside Rb1 (Bai et al., 2021), and Oleanolic acid (Xue et al., 2021), have been reported to affect gut microbiota in metabolic diseases. Therefore, the therapeutic effects of FTZ are postulated to be partly due to the remodeling of gut microbiota. After oral administration, the compounds from FTZ inevitably interact with intestinal flora in the gut. These interplays mainly include (Feng et al., 2019) the following: 1) compounds regulate the composition of gut microbiota, 2) compounds affect the metabolism of gut microbiota, and 3) gut microbiota transform the compounds. In a previous study, serum pharmacochemistry analysis showed that 36 constituents (27 prototype components and nine metabolites originating from FTZ) in FTZ-treated rat serum were identified, which were different from the components directly detected from FTZ extract (44 substances) (Zhong et al., 2012). These results suggest a significant change in FTZ compounds, presumably due to their interaction with gut microbiota.

This study found a profound shift in gut flora composition of HFS-fed rats, characterized by the decreased intestinal microbial richness and diversity. FTZ administration effectively reversed these alterations in both alpha and beta diversities of gut microbial profile. The functional analysis results of the KEGG metabolic pathway showed that the HFS diet significantly upregulated the genes related to carbohydrate metabolism, which may be associated with the increased intake of fructose. FTZ treatment downregulated the genes involved in lipid metabolism and replication and repair, which may be related to the beneficial effects of FTZ on dyslipidemia and renal injury. Additionally, relative abundances of the gut microbiota at phylum and genus levels also demonstrated significant changes. Taken together, these data indicate that FTZ treatment modifies HFS-induced gut dysbiosis.

However, it is still hard to determine whether the alteration of gut microbiota was a concomitant phenomenon or a potential mechanism through which FTZ exerted its antihypertensive effect. Mounting evidence has shown that FMT is a feasible method of confirming the role of gut flora in disease pathology and treatment (Li et al., 2017; Zhou et al., 2019). Hence, we performed FMT to validate if FTZ lowered blood pressure by remodeling intestinal microbiota.

To improve the effectiveness of FMT, most of the gut microbiota was removed with antibiotic pretreatment in all rats. Then, with the diet unchanged, fecal microbial materials obtained from HFS-fed and FTZ-treated HFS-fed donor rats were cross-transplanted to each other and from HFS-fed and NC-fed donor rats to each other, respectively. The results showed that the antibiotic-decreased blood pressure of FTZ-treated flora receivers was further reduced. The antibiotic-decreased richness and diversity of intestinal microbiota were greatly improved, indicating that FMT effectively lowered HFS-induced high blood pressure and restored HFS-induced gut dysbiosis. Interestingly, we noticed that FTZ-treated flora receivers did not exhibit a similar gut microbiota composition with FTZ-treated HFS-fed donor rats but with HFS-fed donor rats and their corresponding receivers. On the contrary, the antibiotic-increased blood pressure of HFS-induced flora receivers was further elevated, and the intestinal microbial richness and diversity were both decreased to the lowest. Additionally, HFS-induced flora receivers displayed a similar gut microbiota composition to HFS-fed donor rats. These results suggest the role of FTZ in gut microbiota alteration and the synergistic effects of the HFS diet and its induced abnormal intestinal microbiota on elevating blood pressure. Furthermore, the results of cross FMT between the NC-fed and HFS-fed rats demonstrated that with the improved intestinal microbial richness and diversity by FMT, both the NC diet and its shaped normal intestinal flora could inhibit the increase of blood pressure induced by HFS-disturbed abnormal intestinal flora and HFS diet, respectively. The comparative analysis of NC-fed and FTZ-treated HFS-fed rats and the two HFS-fed subgroups further confirmed that the HFS diet and the imbalanced gut flora induced by the HFS diet were interdependent in promoting hypertension. In contrast, this effect could be inhibited by a normal gut flora transplantation or a normal diet, respectively.

In conclusion, our data show that FTZ treatment could attenuate HFS-induced hypertension, related metabolic disorders, and renal injury. The antihypertensive effect is partly attributed to restoring gut dysbiosis. Therefore, FTZ holds promise for comprehensively preventing and treating hypertension.

## Data Availability Statement

The datasets presented in this study can be found in online repositories. The names of the repository/repositories and accession number(s) can be found below: NCBI SRA BioProject, accession no: PRJNA810707.

## Ethics Statement

The animal study was reviewed and approved by the Animal Studies Committee of Guangdong Pharmaceutical University.

## Author Contributions

ZC, BY, and ZW performed the experiments. ZC analyzed the data and wrote the manuscript. JG and QZ edited the manuscript. XR contributed to the discussion. JG and QZ conceived and designed the study. All authors contributed to the article and approved the submitted version.

## Funding

This work was supported by the Key Project of the National Natural Science Foundation of China (81830113), National key R&D plan of China “Research on modernization of traditional Chinese medicine” (2018YFC1704200), and Major basic and applied basic research projects of Guangdong Province of China (2019B030302005).

## Conflict of Interest

The authors declare that the research was conducted in the absence of any commercial or financial relationships that could be construed as a potential conflict of interest.

## Publisher’s Note

All claims expressed in this article are solely those of the authors and do not necessarily represent those of their affiliated organizations, or those of the publisher, the editors and the reviewers. Any product that may be evaluated in this article, or claim that may be made by its manufacturer, is not guaranteed or endorsed by the publisher.

## Abbreviations

α-SMA, α-smooth muscle actin; AGT, angiotensinogen; ASV, amplicon sequence variant; AT1, angiotensin type 1 receptor; Cr, creatinine; Cys-C, cystatin C; DBP, diastolic blood pressure; FBG, fasting blood glucose; FMT, fecal microbiota transplantation; FTZ, Fufang-Zhenzhu-Tiaozhi capsule; HPLC, high-performance liquid chromatography; HFS, high fructose and high salt; HR, heart rate; IL-6, interleukin-6; NADPH, nicotinamide adenine dinucleotide phosphate; NC, normal chow; NOX-2, nicotinamide adenine dinucleotide phosphate oxidase-2; NOX-4, nicotinamide adenine dinucleotide phosphate oxidase-4; PCoA, principal coordinate analysis; qRT-PCR, quantitative reverse transcription PCR; RAS, renin–angiotensin system; SBP, systolic blood pressure; SPF, specific pathogen free; TC, total cholesterol; TCM, traditional Chinese medicine; TG, triglyceride; TGF-β, transforming growth factor-β; TNF-α, tumor necrosis factor-α; UA, uric acid.
