# Supplementary material for: Modulation of the Gut Microbiota by Fufang-Zhenzhu-Tiaozhi Capsule Attenuates Hypertension Induced by a High-Fructose and High-Salt Diet
Source: Front Cell Infect Microbiol. 2022 Jun 29;12:854849. doi: 10.3389/fcimb.2022.854849 (PMC9277139; doi:10.3389/fcimb.2022.854849)
Supplement: Supplementary file 1 [file Table_1.docx]

| Gene | Species | Primer sequences |
| --- | --- | --- |
| IL-6 | Rat | Sense：AGTTGCCTTCTTGGGACTGATGTTG |
|  |  | Antisense：GGTATCCTCTGTGAAGTCTCCTCTCC |
| TNF-α | Rat | Sense：CCACGCTCTTCTGTCTACTGAACTTC |
|  |  | Antisense：AGATGATCTGAGTGTGAGGGTCTGG |
| NOX-2 | Rat | Sense：CGAAGACAACTGGACAGGAACCTTAC |
|  |  | Antisense：CCCGACTCTGGCATTCACACAC |
| NOX-4 | Rat | Sense：CCTTTGTGCCTATACTGTGCTGAGAG |
|  |  | Antisense：CATACGGAGTTCCATGACATCTGAGG |
| TGF-β | Rat | Sense：CACTCCCGTGGCTTCTAGTG |
|  |  | Antisense：GGACTGGCGAGCCTTAGTTT |
| α-SMA | Rat | Sense：GATCACCATCGGGAATGAACGC |
|  |  | Antisense：CTTAGAAGCATTTGCGGTGGAC |
| AGT | Rat | Sense：AAGCAGGCGAGGAGGAGCAG |
|  |  | Antisense：GATGGCGAACAGGAACGGACTG |
| AT1 | Rat | Sense：GCTTCAACCTCTACGCCAGTGTG |
|  |  | Antisense：CGAGACTTCATTGGGTGGACGATG |
| β-Actin | Rat | Sense：TGTCACCAACTGGGACGATA |
|  |  | Antisense：GGGGTGTTGAAGGTCTCAAA |
